# Scotch Cottage Hospital Movement

**Published:** 1913-02-01

**Authors:** H. Reid

**Affiliations:** Matron; West Highland Cottage Hospital, Oban


### The Editor's Letter-Box.

### Scotch Cottage Hospital Movement.

*To the Editor of* The Hospital.

Sir,—It was with some surprise that I read the paragraph on p. 450 of last week's Hospital on the Scotch Cottage Hospital Movement. I have not had the opportunity of visiting other cottage hospitals in the Highlands, so do not know at first hand about them. I trust that the enclosed report will show that all cottage hospitals in the Highlands ought not to be included in the rather sweeping charge of mismanagement. Eighteen months ago additions and alterations costing £2,000 were made. This is all paid for. Apologising for troubling you.—I am, faithfully yours, H. Reid, Matron.

West Highland Cottage Hospital, Oban, January 28.

